# Biometric Identification from Human Aesthetic Preferences

**DOI:** 10.3390/s20041133

**Published:** 2020-02-19

**Authors:** Brandon Sieu, Marina Gavrilova

**Affiliations:** Department of Computer Science, University of Calgary, 2500 University Dr. N.W., Calgary, AB T2N1N4, Canada; mgavrilo@ucalgary.ca

**Keywords:** pattern recognition, behavioral biometrics, biometric security, gene expression programming, visual aesthetics, human–machine interactions

## Abstract

In recent years, human–machine interactions encompass many avenues of life, ranging from personal communications to professional activities. This trend has allowed for person identification based on behavior rather than physical traits to emerge as a growing research domain, which spans areas such as online education, e-commerce, e-communication, and biometric security. The expression of opinions is an example of online behavior that is commonly shared through the liking of online images. Visual aesthetic is a behavioral biometric that involves using a person’s sense of fondness for images. The identification of individuals using their visual aesthetic values as discriminatory features is an emerging domain of research. This paper introduces a novel method for aesthetic feature dimensionality reduction using gene expression programming. The proposed system is capable of using a tree-based genetic approach for feature recombination. Reducing feature dimensionality improves classifier accuracy, reduces computation runtime, and minimizes required storage. The results obtained on a dataset of 200 Flickr users evaluating 40,000 images demonstrate a 95% accuracy of identity recognition based solely on users’ aesthetic preferences.

## 1. Introduction

Human–machine interactions rely on human behavior [1]. Behavioral biometrics can prove effective in situations where a person’s mood, emotions, or intent are to be identified. While the majority of biometric research focuses on behavioral biometrics such as voice and gait [2], as well as the enhancement of accuracy through information fusion [3,4], this article presents the most comprehensive study to date on the use of aesthetic-based human traits expressed through human–machine interaction for biometric identification. In the domain of behavioral biometrics, social-behavioral biometrics utilizes a person’s interpersonal interactions, dispositions, and attitudes expressed through online media and communications as features [5,6]. Social network users exhibit many unique features through daily communications [7]. One such feature includes an individual’s visual aesthetic preference, which can be described as the principles or criteria which represent one’s judgment of visual beauty [8]. Research on the identification of individuals based on their visual aesthetics, essentially their visual preferences, emerged very recently [9,10,11]. With knowledge of an individual’s visual preference from a selection of images, corresponding features can be retrieved, which forms the person’s specific visual aesthetic authentication template. This is the basis of visual aesthetic identification. The present research is a significantly extended version of conference paper [12].

As a relatively new domain of research, visual aesthetic identification shows very high potential. Human–machine interaction often includes not only work-related tasks but also online social activities, interactions with family members, recreation and more. Among them, sharing photographs, discussing art, and expressing the liking of posted images are common expressions of human behavior. However, understanding these behaviors has been notoriously difficult due to the numerous visual features that are present in the images. During the creation of user authentication templates, it is important to use the most discriminatory features for classification. In pattern recognition, a feature is a characteristic that represents some data of an observation. Although what are considered good feature characteristics depends heavily on the problem domain, many common techniques aim to improve the efficacy of feature data such as normalization, selection, extraction, and expansion. Highly correlated or otherwise non-discriminatory features can decrease the accuracy and speed of the classifier, with intensity varying depending on the model. The most recent work [11] used 861 original features, reduced to 700 Principal Components (PC) using Principal Component Analysis (PCA). Although the accuracy obtained was higher than in the previous works, a further increase in accuracy and reduction of template generation time were outlined as future challenges. A reduced feature set can lower these times, which is typically done through feature selection or extraction techniques [13].

In this paper, we apply Gene Expression Programming (GEP) to reduce feature dimensionality and increase classifier accuracy in a new identification model for visual aesthetic identification. Gene expression programming is a stochastic, meta-heuristic approach that utilizes structured gene trees to represent generated candidate solutions. The general structure of a GEP model follows other genetic approaches that use the fundamental principles of evolution: selection, mutation, and crossover (also called recombination). Through the use of program generations, unique candidates are generated based on random mutation and crossover with the previous generation to maintain a population of programs [7,14]. The motivation to investigate the benefits of a GEP method for biometric identification originates from the recent successful applications of this method in medicine [15] and physics [16].

A modified GEP-based approach to feature extraction is proposed in this paper to improve aesthetic-based person identification accuracy and reduce enrollment times. This approach transforms the original feature set into a smaller set of complex features through structured program evolution. We establish that these complex features can increase accuracy due to the reduction of noise introduced into the classifier system. A smaller feature vector with higher discriminatory ability also results in fewer computations during classification and a lower amount of memory required to store an authentication template. The proposed approach can be applied to other domains and lead to integration into more complex systems. In proposing such a model, the paper aims to improve the current state-of-the-art visual aesthetic identification and test the efficacy of a GEP approach given a large feature set in this domain. Results surpass the state-of-the-art methods for visual aesthetic identification.

Very preliminary research on this topic has been published as a conference paper [12]. In the current work, additional extensive experimentations on model hyperparameters and analysis have been provided. An adaptive mutation behavior was implemented, which increases the robustness and growth of the overall accuracy of the system. Model performance comparisons between different machine learning algorithms have also been added. Thus, this paper makes the following contributions:

1. A novel visual aesthetic-based identification model is introduced that achieves higher accuracy over the most recently reported results.
2. The investigation of utilizing gene expression programming to construct complex features is conducted for the first time in the aesthetic research domain.
3. The proposed model reduces the dimensionality of the feature data required for identification, which achieves an improvement in both computation speed and system robustness of the visual aesthetic-based identification system.
4. A comparison with the proposed model and previous works is performed on the benchmark dataset.

## 2. Previous Work

The research on visual aesthetics presented in this paper is introduced in the context of social biometrics [5,6], an area of biometrics that understands human behavior and personality traits based on human social network activities. This emerging direction of research was preceded by the development of a new generation of biometric systems based on fundamental modeling principles [17], with a focus on the simulation of biometric information based on real-life models [18]. The majority of research on visual aesthetic identification is very recent, with the initial attempt at identification using visual aesthetics conducted by Lovato et al. [9]. This work used a total of 62 image features from a collected database of 40,000 Flickr images, chosen by 200 users (200 images per user). A lasso-regression model was used to construct the visual aesthetic template to identify users, achieving an accuracy of 14% at rank 1 and 51% at rank 5. Although the accuracy was very low, the work served as a proof of concept and feasibility on the discriminatory usage of basic visual aesthetic features.

A more sophisticated approach was developed by Segalin et al., which utilized a multi-resolution counting grid to categorize similar images [10]. Features were separated into two categories: perceptual features and content features. The experiments resulted in above 70% accuracy at rank 1 on the benchmark dataset collected by the authors of [9].

The more recent work performed by Azam and Gavrilova presented a larger feature set of 861 that consisted of local perceptual features, global perceptual features, histogram of gradients (HOG) features, and content features [11]. The methodology used Principal Component Analysis for feature reduction, which produced 700 principal components from 861 original features. These principal components are complex features generated through linear combinations of the original feature set, sorted by total variance. Using the same database of 40,000 liked Flickr images from 200 users (200 images per user), the system was able to identify users with a rank 1 accuracy of 84.50%, and a rank 5 accuracy of 98%.

In solving this identification problem, the classification techniques used in the previous works consisted of a mixture of traditional machine learning and feature engineering. As a general trend, more features are extracted to improve identification accuracy, with 861 features introduced in the most recent work [11] compared to 62 in the initial work [9]. More sophisticated machine learning techniques have also been shown to improve the system’s ability to capture a person’s aesthetic judgment. However, the introduction of a high number of features can result in a classifier’s diminished performance [19]. Non-discriminative features can be interpreted as noise to a machine learning algorithm that detracts from useful data. This non-discriminative data can also encourage unnecessary computations and storage requirements in a real-world system. Thus, in this work, we propose to utilize gene expression programming for complex feature extraction in order to further improve accuracy and reduce computation time.

The recently introduced GEP methodology has proven highly beneficial in a variety of applications [20]. Like other techniques such as genetic algorithms (GA) or genetic programming (GP), GEP operates using the fundamental principles of genetic evolution. Generally, GEP has been applied to five categories of problems: symbolic regression, classification, automatic model design, combinatorial optimization, and real parameter optimization [21]. GEP leverages the strengths of genetic-based algorithms while retaining simple genetic operations and faster convergence than GP approaches in complex optimization problems [22]. Thus, we hypothesize that GEP will be highly suitable for the aesthetic identification problem. The benefits of GP and GEP for feature construction are numerous, with solution trees used for both single and multi-dimensional feature construction [23]. Single dimensional feature constructions using GP compresses many features into one complex feature encoded by a single GP tree [24]. Though performance is favorable in certain scenarios, one complex feature may not be enough to accurately discriminate between classes in a problem with high complexity. Thus, multi-dimensional approaches have been proposed that include using many evolutionary generations to implement a unique function which designates a new feature in each subtree [25]. Although the application of GEP on large feature dimensionality has been explored, it was never considered for the behavioral biometric classification problem.

## 3. Methodology

### 3.1. Overview

**Figure 1:**
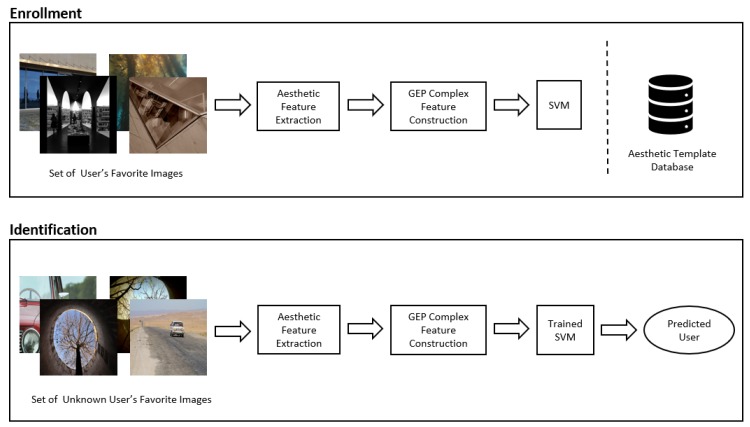
Enrollment and identification phases of the visual aesthetic-based identification system.

The proposed system is a visual aesthetic-based identification system that utilizes GEP-based feature construction and an SVM to classify individuals. Given a collection of favorite images from a user, the model first extracts pertinent perceptual and content features. This high dimensionality feature vector is then inputted into the GEP constructed tree structure to reduce dimensionality, before being used to train the SVM in the enrollment phase. During identification, the features from the set of images from an unknown user will be extracted and transformed using the same GEP structure to a lower dimensionality. The trained SVM will then be consulted and the stored template of the closest user will be returned as the prediction. A figure illustrating the enrollment and identification phases of the system is shown in Figure 1.

**Figure 2:**
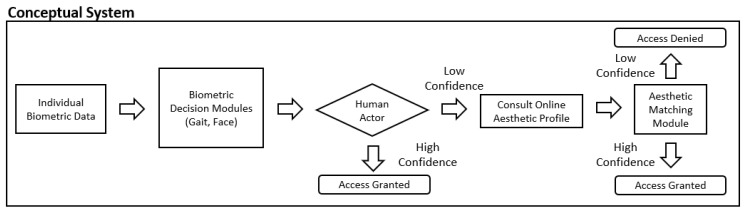
The conceptual flowchart of a human-assisted decision-making process.

The illustration of a conceptual flowchart using the proposed aesthetic identification system is shown in Figure 2. The figure depicts a fully developed aesthetic matching system that can be used to reinforce or assist a human’s decision when there is low confidence. After using a person’s physiological traits (such as gait or face) for biometric identification, if needed, the human actor can consult the aesthetic identification module to aid in making a final decision. The individual’s online aesthetic profile is then used for authentication with the template database. In comparison to other traditional biometric modalities, aesthetic identification is non-intrusive and widely accessible. This can prove effective when the aesthetic matching module is used as a component of a deeper multimodal security system. Note that this is only one of the possible usage scenarios for the proposed system. Other applications in retail, online shopping, and remote communications are also possible.

### 3.2. Feature Extraction

The database of 200 users labeled to 200 of their favorite images is used [9], with a total of 40,000 images and size of 6.35 GB. First, the set of favorite images for each user is processed by extracting a predetermined number of features. These features constitute the original feature set. The features are categorized into three general groups: global perceptual and content features, local perceptual features, and histogram of oriented gradients (HOG) features. This categorization is made based on the feature categories recommended in [9,10,11].

**Figure 3:**
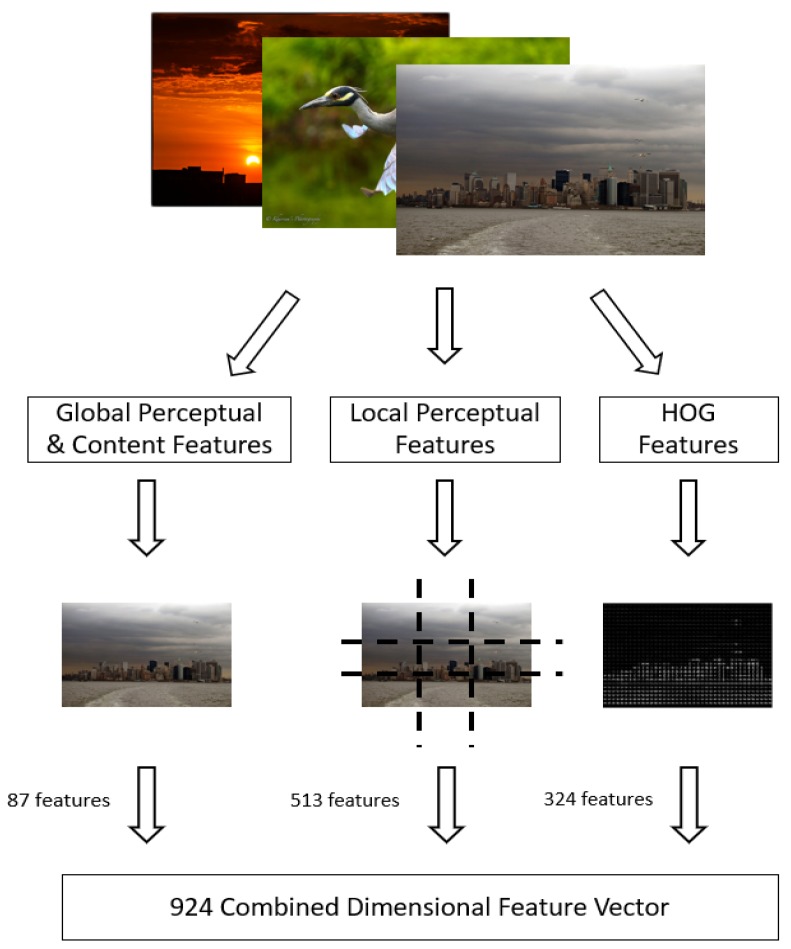
The categorization and count of features extracted for each image.

Perceptual features describe direct properties of the image such as mean hue, saturation, and value (HSV), colorfulness, and entropy of pixel intensities. The further distinction between global and local perceptual features is the applied space—global perceptual features are taken from the entire image, while local perceptual features are taken from each of nine evenly split partition cells of the image in a grid fashion. Content features describe the count and average area of detected faces and objects in the image (bikes, birds, boats, bottles, buses, cars, cats, chairs, dogs, horses, motorbikes, people, planes, and tables). Lastly, the HOG features are generated using gradient values returned from point locations of the image based on the corresponding angle and magnitude maps. All feature values are normalized with zero mean/unit standard deviation and largely operate on the pixel properties (color space) of the image. The feature categorization is shown in Figure 3.

After the concatenation of these features into a combined high dimensional feature vector, it is preprocessed before undergoing feature extraction. The GEP module takes as input the original feature set of size 924 and performs various logical and arithmetic recombinations to produce an evolving complex feature set. A large portion of the implementation is in the GEP module, with the classifier system serving as a wrapper function to direct the evolutionary fitness of each generated feature. Each candidate solution is evaluated using the classifier prediction accuracy with 5-fold random split cross-validation.

After a pre-determined number of generations, the GEP module outputs a lower-dimensional feature vector of complex features which is subsequently used to generate visual aesthetic templates for every user in the training set. A similar process is followed for user identification, where the complex feature score for the user to be identified is calculated and compared to the stored templates in the template database. The user corresponding to the stored visual aesthetic template generated at enrollment which matches most with the provided template is chosen as the system prediction.

### 3.3. Gene Expression Programming Based Feature Construction

**Figure 4:**
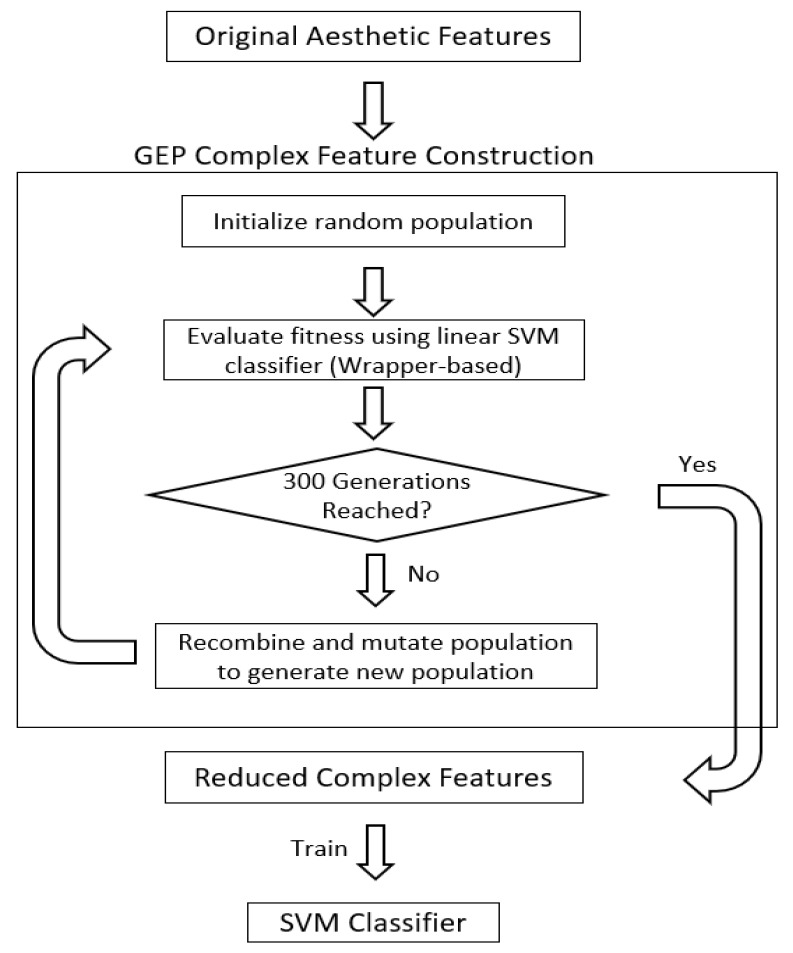
The flowchart for the Gene Expression Programming method for feature selection and classification.

As a genetic-based algorithm, the genetic operators of selection, mutation, and recombination are used to generate new complex features. These complex features take the form of tree-based structures, where leaf nodes are called terminals and branch nodes are called functions. Terminals are selected from the pool of features from the original feature set, while functions are selected from a pool of available mathematical and logical functions. A candidate solution is called a chromosome, which is composed of subtrees called genes. Collectively, a generation is composed only of a pre-determined number of chromosomes called the population, which shifts every iteration of the algorithm simulating evolution. A small process map of the GEP algorithm is shown in Figure 4, which is a common procedure of many genetic-based approaches.

Each of these tree expressions is evaluated given the corresponding values in the original feature set to create a transformed feature vector. To store a multi-dimensional complex feature, an individual complex feature is defined as a gene subtree of the candidate chromosome. Each gene encodes one complex feature, with the combined feature vector represented as a multi-dimensional vector of its composite gene values over all observations. These candidate solution vectors are the structures that are ultimately optimized for accuracy using classifier accuracy as a fitness function.

#### 3.3.1. Model Parameters

Most model parameters of GEP affect the genetic operation phases directly. The number of genes and the head length afforded to each chromosome is determined at the construction of the model, which is fixed throughout the program after definition. This determines the overall complexity and number of generated features. A gene number of 150 and a head length of 30 was used. The values of 150 complex features and 30 head length were determined empirically, showing the most sustained improvement and faster convergence to the solution. These values are subject to variation between problem domains as the number of original features, the complexity of the problem and the nature of the problem are vital considerations for determining the complex feature structure.

The population of each generation is also an important consideration, where a small population can result in higher probabilities of model stagnation and a large population results in longer generation times on average. A balance between computational load and model efficacy was sought in this research. A population of 30 was used, which proved to be a large enough population to avoid most cases of homogeneity among the active population while taking time and computational restraints into consideration.

**Figure 5:**
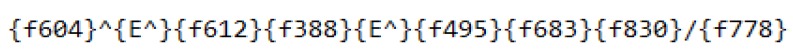
Example of a string representation of a feature.

The initialization of the starting population is randomly selected by drawing within the pool of available terminals and functions. Although there is a scaled fitness selection method, the initial population will always be independently random, due to the absence of fitness direction. The head and gene counts of the candidates are fixed, meaning that, even at initialization, all complex features generated will exhibit syntactic correctness (there will not be any uncharacteristically unstructured candidates, for example). The pool of available terminals and functions used in the project are included in Table 1. An example of the string representation for a candidate feature is depicted in Figure 5.

#### 3.3.2. Fitness

The fitness of every complex feature vector in the population is evaluated every generation. A wrapper-based approach to fitness is applied, where the complex feature vector is tested with the classifier for every candidate. The fitness value is produced through 5-fold cross-validation using random splitting. The stratified and randomized test/training set pairs avoid the selection of an overfitted set of complex features that can only successfully classify for very limited cases. Any candidates that are unviable due to mathematical/logical errors are automatically assigned a fitness value of 0 and effectively discarded due to the nature of evolutionary bias. The combination that generates the highest classification accuracy is then chosen to persist to the next generation as a simple form of elitism. The best candidate of the population will not be mutated or recombined (but can be recombined with) to preserve its discriminatory ability.

#### 3.3.3. Termination Condition

The termination condition used in the model was 300 generations. The time required per generation increases significantly based on training and testing sample dimensionality; therefore, the number of generations in this implementation may be smaller than other GEP implementations. Maintaining 300 generations proved to capture a sufficient period for growth and usually led to indicators of convergence to a solution. For larger user datasets, the number of generations will likely need to increase, as it will take the system more evolutionary generations to converge on a larger solution space.

#### 3.3.4. Mutation

Mutation in the GEP implementation operates by selecting a random index with probability based on the pre-determined mutation rate. This implementation uses a base adaptive mutation rate that decreases proportionally to classifier accuracy, with an increasing mutation rate of 0.05 per generation without improvement compared to the last best accuracy. When an improvement is experienced, the mutation rate will return to the base adaptive mutation rate.

A base adaptive mutation rate allows for the system to adjust the minimum amount of mutations every generation. The intuition behind this is to restrict the explorable solution space as smaller changes to the existent solution are required. By allowing the mutation rate to increase above the base adaptive rate during periods of non-improvement, the model increases the explorable solution space to potentially escape local maxima. This will allow the model to restrict and release the solution space by manipulating the probability of random changes to every node of every solution tree, which produces a more robust searching model. This is especially significant when the population has reached an over homogeneous state or has difficulty converging onto a smaller solution space.

Let MR be the mutation rate, $n_{C}$ be the current generation count, $n_{B}$ be the generation count with the best accuracy, and *A* be the accuracy as a decimal value. Then, the computation is carried out as:

(1) $$\begin{array}{c} MR\left(n_{C}\right)=\left\{\begin{array}{cc} \frac{\left(1-A\left(n_{C}\right)\right)}{10} & \mathrm{if}\;A\left(n_{C}\right)>A\left(n_{B}\right)\\ \frac{\left(1-A\left(n_{C}\right)\right)}{10}+\left(0.05*\left(n_{C}-n_{B}\right)\right) & \mathrm{if}\;A\left(n_{C}\right)\leq A\left(n_{B}\right) \end{array}\right. \end{array}$$

The value of the denominator of 10 was used to restrict the mutation rate to values within a range of 0.0 to 0.1, or 0% to 10%. Regardless of the initialization of the population, the best solution at any given generation is typically higher than 50%, as even non-optimal complex features carry some discriminatory ability. The increment of 0.05 acts as an increased weight to the mutation rate, which was determined empirically to ensure the impact is significant yet controlled. This increment is then multiplied with the period of non-improvement, denoted by $n_{C}-n_{B}$.

#### 3.3.5. Crossover

Crossover is the random exchange of a set of genes between two candidate features. The number and location of these exchanges are random and can occur multiple times throughout a single gene crossover. This implementation uses a unique variable uniform, one-point, and two-point crossover rates based on current best classifier accuracy. A uniform gene crossover is a default rate at which every node of either candidate solution can be chosen, while an *n*-point crossover denotes n+1 regions where all nodes of one candidate are chosen. The variable crossover rate increases as the proposed complex feature vector inputted into the classifier gains accuracy. This allows a higher probability of the candidates in the population to converge more into a smaller solution space. Feature sets with discriminatory complex features at this stage are encouraged to crossover with other capable candidates, which aims to increase the chances of convergence on a solution.

Let $CR_{0}$ be the uniform crossover rate, $CR_{1}$ be the one-point crossover rate, $CR_{2}$ be the two-point crossover rate, and *A* be the accuracy as a decimal value. Then, the computation is carried out as:

(2) $$\begin{array}{cc} \; & CR_{0}=\frac{A}{4} \end{array}$$

(3) $$\begin{array}{cc} \; & CR_{1}=2CR_{0} \end{array}$$

(4) $$\begin{array}{cc} \; & CR_{2}=2CR_{0} \end{array}$$

The denominator of 4 was used to ensure the crossover rate did not exceed 0.25 but maintained a high chance per node to be exchanged. In addition, the one-point and two-point crossover rates increase in proportion to the uniform crossover rate. This allows for an increased rate of diverse crossover, which contributes to a more varied resulting population.

#### 3.3.6. Selection

The selection phase collects the resultant, evolved population and iterates back to the evaluation phase. During this stage, the GEP model uses simple elitism by automatically carrying and saving the best candidate from the previous generation into the next. This maintains at least one complex feature combination with the best current accuracy, allowing the other candidate solutions in the next generation the opportunity to crossover. A bias is applied through a scaled roulette wheel that favors candidates with higher accuracy. These solutions will be allowed an increased chance of reproduction with other solutions.

### 3.4. SVM-Based Identification

The evaluation of the fitness of each candidate solution vector is handled by a linear support vector machine (SVM) classifier. Each evaluation score is generated from the mean of 5-fold cross-validations on the train and test sets, with a random splitting of sample indices. When performing multi-class prediction, the classifier adopts the One-Versus-Rest (OVR) approach by constructing a binary classifier for each class, with samples from that corresponding class marked as positive, while all other classes are negative. The combination stage takes the classifier result with the highest confidence score. Preprocessing is vital due to the potential distribution of the complex features produced. The complex features are preprocessed through standardization and a power transform to resemble more Gaussian-distributed data. Hyperparameters for the classifier favored classifier accuracy and speed of classification, as the speed of the classifier is directly correlated to the efficacy of the gene expression model. A reduced classification time can allow for more extensive feature combinations, a higher candidate population, and additional generations. Classifier hyperparameters are evaluated before the GEP implementation.

## 4. Experimentation and Results

The classification system was implemented in Python 3.5, using the open-source PyGEP 3.5 port library for the base GEP framework. All experiments were run on a Windows distribution using an Intel Core i7-8700 @ 3.20 GHz CPU and an Nvidia GeForce GTX 1080 GPU. Six cores, 12 logical processors, and 16 GB RAM are available to the machine.

The system is tested on a 40,000 Flickr benchmark dataset consisting of 200 users each with 200 labeled favorite images [9]. An overall rate of repeated images chosen by the different users is 0.05%. A large feature vector was extracted and precomputed from the local, perceptual, content and HOG features. The images are split into a 50–50 distribution of testing and training sets, with different combinations of these images organized for 5-fold cross-validation. Each image set is composed of 100 images. The 5-fold cross-validation is applied within the image set level, where each fold shuffles the choice of the images used within the 100 image sets. This ensures that each test and training set is different between folds.

To evaluate the strength of the computed complex features, the GEP approach was used with a gene number or complex feature count of 150 on the same classification problem. A termination condition of 300 generations was used, as the improvement of the produced solution was found to become stable after this generation count.

The experiments were designed to verify the system’s ability to perform highly accurate identification reliably and efficiently. The experiments begin with the testing of the optimal generation and gene count. This configuration is then used to compare the performance of different classifiers, where SVM produces the best result. Performance metrics for the system including the cumulative matching characteristic, receiver operating characteristic, and the false positive/negative graphs are shown. These results are compared to the current state-of-the-art methods, with the proposed system outperforming the most recent published model in [11] and its reimplemented result. Lastly, a final comparison shows that the GEP model outperforms other common dimensionality reduction techniques for the visual aesthetic-based identification problem.

**Figure 6:**
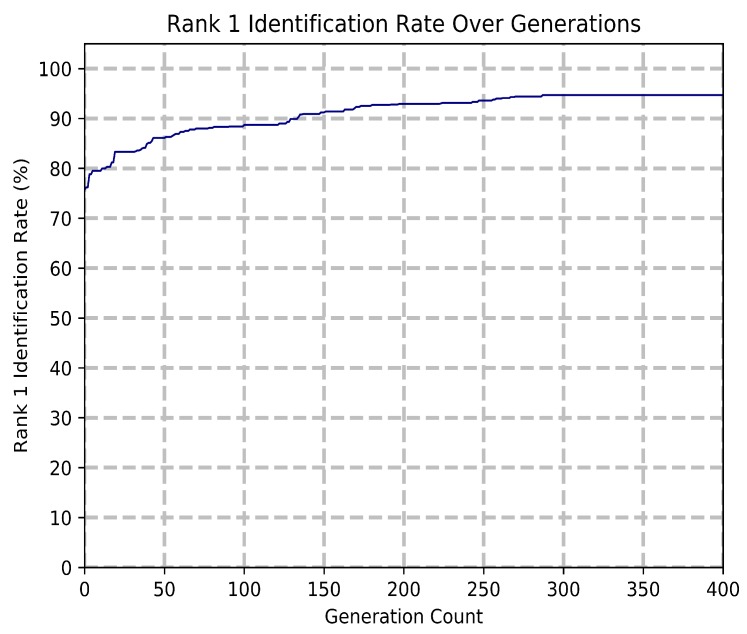
Model accuracy over a period of 400 generations.

Figure 6 shows a graph of the performance of the system using the SVM classifier for a period of 400 generations. The initial best candidate has a rank 1 identification accuracy of 76%, which increases to 93% based on steady improvement observed from generation 0 to 200. The intervals of improvement grow smaller between generation 200 and 300, reaching an accuracy of 95%. Beyond generation 288, no further improvement to the system accuracy can be observed. This is indicative of the GEP module being unable to find a better solution within a heavily restricted solution space. Even after 100 generations of no improvement, the system with a high variable mutation rate remains unable to find a better solution in the less restricted solution space. A maximum has been reached, with the probability of both escaping and finding a better solution low. Therefore, a baseline generation count of 300 was used.

**Figure 7:**
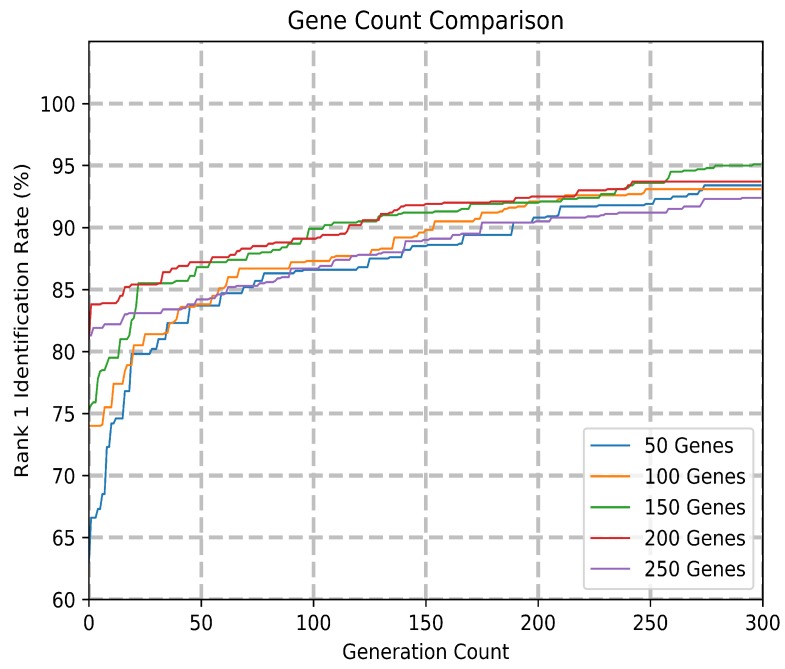
Comparison of model accuracy over 300 generations with various gene counts.

The choice of gene count was determined empirically, with the results of the experiments shown in Figure 7. The rank 1 accuracy comparison with the different gene counts is available in Table 2. Since each gene subtree encodes a complex feature, the gene count parameter determines the number of complex features in the resulting feature vector. A higher feature count allows for an expanded solution space with more trainable parameters but is prone to higher generation count required and difficulty in convergence. A lower feature count is a primary objective of feature dimensionality reduction, though a feature count that is too low can be unable to capture sufficient discriminatory information between classes. From these tests, a gene count of 150 proved to result in a system with the highest accuracy at generation 300.

**Figure 8:**
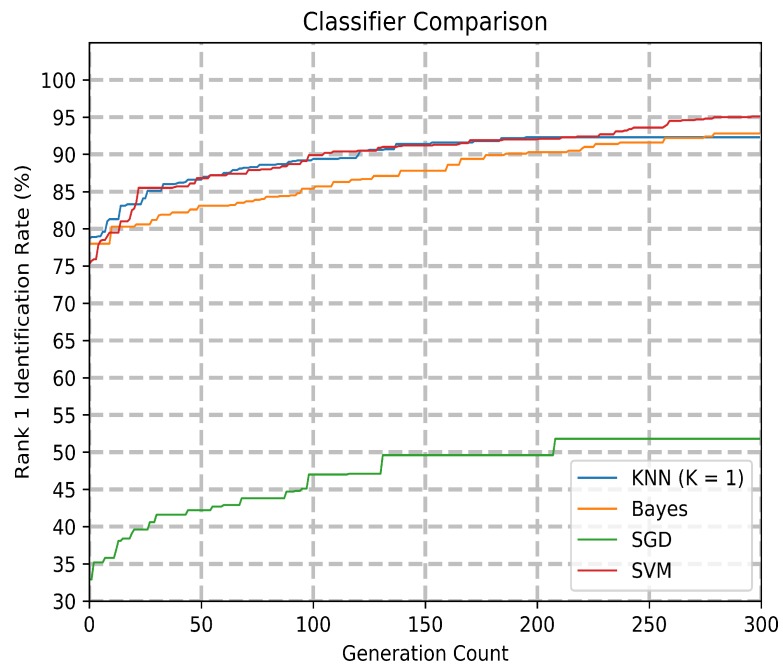
Model accuracy comparing different classifiers.

A comparison of the different classifiers is shown in Figure 8. The rank 1 accuracy comparison with the different classifiers is available in Table 3. K-Nearest Neighbor (KNN), Naive Bayes, Stochastic Gradient Descent (SGD) Classifier, and Support Vector Machines are compared, with SVM showing the highest rank 1 identification at 95.1%. The tolerance for the SGD and SVM classifiers were both set to 0.001, with the SVM set to the linear kernel with a regularization parameter of 0.25. Each classifier’s decision boundaries can influence the gene expression programming module with every evaluation, with an effect on both accuracy and model runtime. SVM was found to be the best performing classifier.

**Figure 9:**
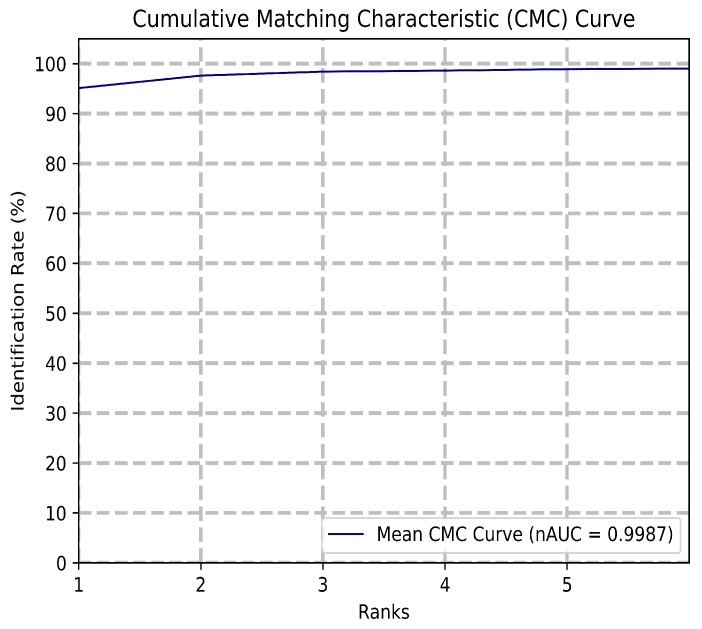
Cumulative matching characteristic curve for the first six ranks, and nAUC for all 200 ranks.

The Cumulative Matching Characteristic (CMC) curve is shown in Figure 9, which displays the proposed classification system’s accuracy across rank 1 to rank 5 recognition rates. A CMC curve is a common indication of classifier accuracy, as it displays the correctness at each rank of prediction. A rank N recognition rate is the probability that the correct prediction is chosen among the top N matches. The normalized Area-Under-the-Curve (nAUC) of a CMC curve is a performance metric that gauges accuracy over all ranks for a specific classifier system, where a value of 1 is ideal accuracy. The system achieves a normalized AUC of 0.9987 among all 200 classes, with a rank 1 recognition of 95.1% and a rank 5 recognition of 98.9%.

Given the prediction probabilities for the test samples, a CMC curve is constructed by calculating the rank N recognition rate for each rank. As the system identifies individuals, there are 200 ranks. This is depicted in the following formula:

(5) $$\begin{array}{cc} \; & P_{N}=\mathrm{Correct}\;\mathrm{identifications}\;\mathrm{within}\;\mathrm{top}\;N\;\mathrm{matches}\;T_{e}=\mathrm{Size}\;\mathrm{of}\;\mathrm{testing}\;\mathrm{sample} \end{array}$$

(6) $$\begin{array}{cc} \; & Rank_{n}Accuracy=\frac{P_{n}}{T_{e}} \end{array}$$

**Figure 10:**
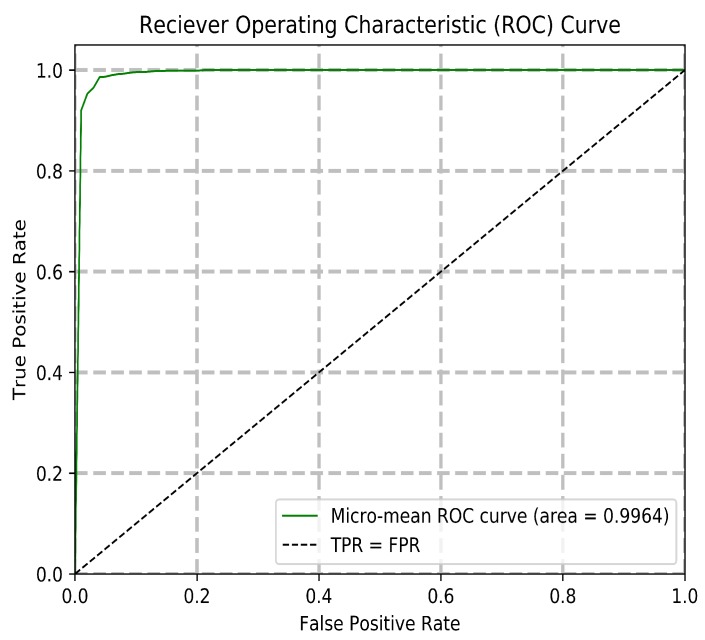
Receiver operating characteristic for all 200 users.

**Figure 11:**
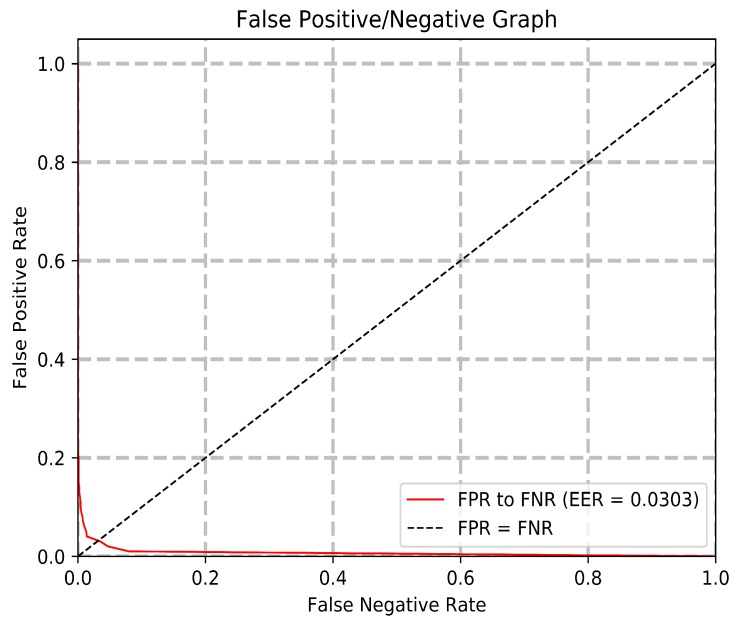
False positive/negative rates for all 200 users and calculated equal error rate.

The Receiver Operating Characteristic (ROC) curve in Figure 10 shows the relation between a system’s true positive rate over false positive rate. A high true positive rate and area-under-the-curve are indicative of a more robust model with less verification error. The false positive/negative graph in Figure 11 shows the false positive rate (Type I error) over false negative rate (Type II error), along with the equal error rate (EER). A false positive corresponds to the incorrect acceptance of an unknown user, and a false negative corresponds to the incorrect rejection of an enrolled user. A lower equal error rate is desired for a biometric system, as it is less prone to making both types of verification error. The model exhibits a ROC area-under-the-curve of 0.9964, with an equal error rate of 0.0303.

The equal error rate is the point at which the false positive rate and the false negative rate intercept. In the context of this multiclass problem, a micro-mean approach is taken, where the ROC and EER metrics were averaged for all binary class scenarios using the one-versus-rest strategy. The relationship between the ROC and EER metrics are depicted in the following formula:

(7) $$\begin{array}{cc} \; & TruePositiveRate\left(TPR\right)=\frac{\mathrm{True}\;\mathrm{Positives}}{\mathrm{False}\;\mathrm{Negatives}+\mathrm{True}\;\mathrm{Positives}} \end{array}$$

(8) $$\begin{array}{cc} \; & FalsePositiveRate\left(FPR\right)=\frac{\mathrm{False}\;\mathrm{Positives}}{\mathrm{True}\;\mathrm{Negatives}+\mathrm{False}\;\mathrm{Positives}} \end{array}$$

(9) $$\begin{array}{cc} \; & TrueNegativeRate\left(TNR\right)=\frac{\mathrm{True}\;\mathrm{Negatives}}{\mathrm{True}\;\mathrm{Negatives}+\mathrm{False}\;\mathrm{Positives}} \end{array}$$

(10) $$\begin{array}{cc} \; & FalseNegativeRate\left(FNR\right)=\frac{\mathrm{False}\;\mathrm{Negatives}}{\mathrm{False}\;\mathrm{Negatives}+\mathrm{True}\;\mathrm{Positives}} \end{array}$$

(11) $$\begin{array}{cc} \; & EER=\mathrm{Equal}\;\mathrm{Error}\;\mathrm{Rate}=FNR_{N}\cap FPR_{N} \end{array}$$

### Analysis

Three of the referenced models are compared to the proposed model, with data shown in Table 4. The experiments were conducted on the same image dataset initially collected by Lovato et al. [9]. The overall classification accuracy of the system increases as classification accuracy is used to optimize an initially random complex feature set, surpassing the original accuracy seen in [11] while using only 150 computed complex features generated over 300 generations. The 700 principal components used in [11] is also far higher than the computed feature size in the proposed model. An improvement of 81.1% percent was achieved over the original 2012 work [9], 22.1% percent over 2014 work [10], and 11.1% over the most recent work [11] at rank 1. Rank 5 results were also improved. In addition, the proposed model effectively used 550 fewer features in the resultant feature vector than the most recent work. The GEP implementation offers high accuracy in these test scenarios by constructing highly discriminative features that are not bound by only linear combinations of the original feature set. Using a similar original feature vector size as [11], the usage of GEP and the novel customization of its genetic operators have heavily reduced feature dimensionality while increasing the discriminative ability of the model. The proposed model also surpasses the rank 1 and rank 5 recognition rates of other state-of-the-art works operating on the same dataset. The dataset used is the largest benchmark dataset for person identification using visual aesthetics.

As more improvements are made throughout the generations, the growth in classifier accuracy decreases as it becomes more difficult to generate further discriminating features without overlap. More generations are required to fine-tune smaller changes to the feature trees due to the random nature of the evolutionary operations. It is also important to consider 300 generations as a low count for typical genetic-based algorithms. The largest expense to the model is the evaluation of every individual complex feature vector, which takes on average under 10 s. Each generation requires at most *n* evaluations, along with evaluations required for refitting the classifier. Further tests with higher generation count, higher population size, or additional complex features can potentially increase accuracy rates further.

In addition, the state-of-the-art approach presented in [11] has been fully reimplemented in MATLAB and compared against the proposed model on the same machine, with results shown in Table 5. The method in [11] uses PCA to generate 700 principal component features and lasso-regression for classification.

The proposed model is shown in Table 6 to have much higher rank 1 and rank 5 recognition rates when compared to the reimplementation, with an improvement of 14.5% and 1.6%, respectively. This is indicative of a more accurate classification system, which is a product of the complex features constructed by the GEP module along with the linear SVM classifier.

In addition, the proposed model is compared with the most recent state-of-the-art methods in Table 5. The amount of time required to generate and test the aesthetic template of all 200 users given the feature vector is significantly lower with the proposed model at 6.71 s. This difference is likely due to the efficiencies of the linear SVM in classifying this distribution of observations and the smaller dimensions of the complex feature vector compared to the PCA feature vector. The smaller complex feature vector when stored only requires 150 megabytes (MB), which is much less than the 511 MB required for the reimplemented approach. The proposed model has higher rank 1 accuracy when compared to the recent conference results by 1%, with similar time and memory requirements.

Additional tests are shown in Table 7 that display the performance of the classification system with different types of dimensionality reduction techniques. All techniques were set to output a reduced dimensionality of 150 from the original 924 feature vector for accurate comparison. Independent component analysis discovers statistically independent components of the feature set through maximizing rotated component non-Gaussanity. Factor analysis searches for underlying latent variables that describe observed variables by considering joint variations or factors. Lastly, principal component analysis extracts linear combinations of the feature vector that explain the most variance. It is shown that the proposed GEP module reduces the components while also increasing the accuracy of the original feature set over other existent dimensionality reduction techniques. When applied on the 924-feature set, the GEP module reduced dimensionality to 150 components while having a 16.2% increase in rank 1 accuracy over using PCA.

Summarizing the performed experiments, both the rationale and the performance of the system have been validated. First, it was discovered that a gene count of 150 performed the best among a set range of different gene counts. This gene count translates to 150 complex features, as each gene subtree encodes one complex feature. Using 150 complex features, the GEP model outperformed common dimensionality reduction techniques such as Independent Component Analysis, Factor Analysis, and Principal Component Analysis when applied to the same feature space. Different classifier configurations are tested with the model, with a Support Vector Machine-based classification outperforming K-Nearest Neighbor, Naive Bayes, and Stochastic Gradient Descent. The Cumulative Matching Characteristic curve establishes the system’s consistently accurate performance across ranks and a normalized Area-Under-the-Curve of 0.9987. A Receiver Operating Characteristic Area-Under-the-Curve of 0.9964 and an Equal Error Rate of 0.0303 proves the system’s robustness and low chance of misidentification. The proposed system achieves a rank 1 accuracy of 95.1% and a rank 5 accuracy of 98.9%. The time and memory required for this system are much lower than the reimplementation of the most recent state-of-the-art method. The results prove that the proposed model outperforms the state-of-the-art visual aesthetic identification methods in accuracy, speed, and storage required.

## 5. Conclusions and Future Works

This article presents the most comprehensive study to date on the use of aesthetic-based human traits expressed through human–machine interaction for biometric identification. It answers the research question of whether a new algorithm can be developed based on gene expression programming that can significantly outperform previous approaches for aesthetic-based human identification. The research demonstrated on a large dataset through extensive experimentation that GEP is an effective method for feature extraction in visual aesthetic identification and that the proposed model reduces the number of features required for identification while also increasing the overall recognition rate. The method reduced the dimensionality of the large original feature set while achieving rank 1 and rank 5 accuracies of 95.1% and 98.9%, respectively. The time and memory requirements have also been shown to be lower than in the previous methods.

This study opens new avenues in harvesting rich information provided during human–machine interaction, where human aesthetics plays a vital role in selecting favorite images. A more thorough investigation into the model implementation may allow the opportunity for further optimizations in system performance through the use of extensive parallelism. In addition to system complexity, the selection, mutation, and crossover strategies can be further developed to increase the robustness and the accuracy of the converged solution. On a broader level, the exploration of alternative ways of human–machine interaction based on other aesthetics, such as music and touch, can be performed for the development of more comprehensive human–machine interfaces as well as more versatile security systems.

## Figures and Tables

**Table 1:** The pool of available node values in the GEP complex feature tree.

Category	Arity	Representation
Terminals	0	$\left\{f_{0},f_{1},...,f_{n}\right\}$
Constants	0	{0,1,π,e}
Power	2	{^}
Arithmetic	2	{+,−,*,/,%}
Comparison	2	{=,!=,<,>,<=,>=}

**Table 2:** Summary comparison of rank 1 accuracy between various gene counts used in the system.

Gene Count	Rank 1
50	93.4%
100	93.1%
**150**	**95.1%**
200	93.7%
250	92.4%

**Table 3:** Summary comparison of rank 1 accuracy between different classifiers used in the system.

Model	Rank 1
KNN	92.3%
Naive Bayes	92.8%
SGD	51.8%
**SVM**	**95.1%**

**Table 4:** Summary comparison of rank 1 accuracy, rank 5 accuracy, and number of features with state-of-the-art works.

Model	Rank 1	Rank 5	Number of Features
Lovato et al. (2012) [9]	14%	51%	62
Segalin et al. (2014) [10]	73%	92%	111
Azam & Gavrilova (2017) [11]	84%	98%	861 (700 PC)
**Proposed GEP Model**	**95.1%**	**99%**	**924 (150 CF)**

**Table 5:** Comparison of model time.

Model	Rank 1	Time	Memory
Reimplemented [11]	80.6%	367 s	511 MB
Sieu & Gavrilova (2019) [12]	94.1%	6.24 s	150 MB
**Proposed GEP Model**	**95.1%**	**6.71 s**	**150 MB**

**Table 6:** Comparison of reimplemented [11] and the proposed method.

Model	Rank 1	Rank 5
Reimplemented [11]	80.6%	97.3%
**Proposed GEP Model**	**95.1%**	**98.9%**

**Table 7:** Comparison of different dimensionality reduction techniques and the proposed GEP module.

Dimension Reduction Method	Rank 1	Features
Independent Component Analysis (ICA)	64.4%	150
Factor Analysis (FA)	76.4%	150
Principle Component Analysis (PCA)	78.9%	150
**Proposed GEP Model**	**95.1%**	150

## Abbreviations

The following abbreviations are used in this manuscript:

CF	Complex Features
CMC	Cumulative Matching Characteristic
EER	Equal Error Rate
FA	Factor Analysis
GA	Genetic Algorithms
GEP	Gene Expression Programming
GP	Genetic Programming
HOG	Histogram of Oriented Gradients
HSV	Hue, Saturation, Value
ICA	Independent Component Analysis
KNN	K Nearest-Neighbour
MB	Megabytes
nAUC	Normalized Area-Under-the-Curve
OVR	One-Versus-Rest
PCA	Principle Component Analysis
PC	Principal Components
ROC	Receiver Operating Characteristic
SGD	Stochastic Gradient Descent
SVM	Support Vector Machine
